# Metabolic Inactivity and Re-awakening of a Nitrate Reduction Dependent Iron(II)-Oxidizing Bacterium *Bacillus ferrooxidans*

**DOI:** 10.3389/fmicb.2019.01494

**Published:** 2019-07-03

**Authors:** Guo-Wei Zhou, Xiao-Ru Yang, Regin Rønn, Jian-Qiang Su, Li Cui, Bang-Xiao Zheng, Yong-Guan Zhu

**Affiliations:** ^1^Key Laboratory of Urban Environment and Health, Institute of Urban Environment, Chinese Academy of Sciences, Xiamen, China; ^2^State Key Laboratory of Urban and Regional Ecology, Research Center for Eco-Environmental Sciences, Chinese Academy of Sciences, Beijing, China; ^3^Department of Biology, University of Copenhagen, Copenhagen, Denmark; ^4^Faculty of Biological and Environmental Sciences, University of Helsinki, Lahti, Finland; ^5^College of Advanced Agricultural Sciences, University of Chinese Academy of Sciences, Beijing, China

**Keywords:** nitrate-dependent Fe(II)-oxidization, *Bacillus ferrooxidans*, metabolic inactivity, re-awakening, Raman spectroscopy, PMA assisted 16S rRNA quantification

## Abstract

Microorganisms capable of anaerobic nitrate-dependent Fe(II) (ferrous iron) oxidation (ANDFO) contribute significantly to iron and nitrogen cycling in various environments. However, lab efforts in continuous cultivation of ANDFO strains suffer from loss of activity when ferrous iron is used as sole electron donor. Here, we used a novel strain of nitrate-dependent Fe(II)-oxidizing bacterium *Bacillus ferroxidians* as a model and focused on the physiological activity of cells during ANDFO. It was shown that *B. ferrooxidans* entered a metabolically inactive state during ANDFO. *B. ferrooxidans* exhibited nitrate reduction coupled with Fe(II) oxidation, and the activity gradually declined and was hardly detected after 48-h incubation. Propidium monoazide (PMA) assisted 16S rRNA gene real-time PCR suggested that a large number of *B. ferrooxidans* cells were alive during incubation. However, ^2^H(D)-isotope based Raman analysis indicated that the cells were metabolically inactive after 120-h of ANDFO. These inactive cells re-awakened in R2A medium and were capable of growth and reproduction, which was consistent with results in Raman analysis. Scanning electron microscopy (SEM) observation and x-ray diffraction (XRD) revealed the formation of Fe minerals in close proximity of cells in the Fe(II)-oxidizing medium after Fe(II) oxidation. Overall, our results demonstrated that continued ANDFO can induce a metabolically inactive state in *B. ferrooxidans*, which was responsible for the loss of activity during ANDFO. This study provides an insight into the ANDFO process and its contribution to iron and nitrogen cycling in the environments.

## Introduction

An important nitrogen turnover process of nitrate reduction is closely linked to Fe(II)-oxidation under anoxic conditions, contributing greatly to nitrogen and iron cycling in the environment (Weber et al., 2006; Borch et al., 2010; Melton et al., 2014). Microorganisms capable of performing ANDFO have been known for more than two decades (Straub et al., 1996), and they use nitrate (NO_3_^–^) and intermediates or end products of denitrification as well as of dissimilatory nitrate reduction (including NO_2_^–^, NO, and N_2_O) as electron acceptors with the iron (II) as electron donor (Weber et al., 2006). Most of the isolated ANDFO strains grow mixotrophically, including *Acidovorax* sp. strain BoFeN1, *Acidovorax* sp. strain 2AN, *Thiobacillus denitrificans*, *Cupriavidus necator*, and *Desulfitobacterium frappieri* strain G2, and need an organic co-substrate for continuous Fe(II) oxidation and growth (Shelobolina et al., 2003; Chakraborty et al., 2011; Verbaendert et al., 2011; Beller et al., 2013; Klueglein and Kappler, 2013). Hence, continuous cultivation with ferrous iron as sole electron donor is impossible for most of these ANDFO bacteria (Kappler, 2005). After several successive generations, these strains cannot oxidize ferrous iron in absence of an organic substrate. However, little is known about this phenomenon; e.g., the physiological activity of the cells during ANDFO has not been described and it is not known whether iron oxidation may induce a metabolically inactive state.

Currently, potential approaches to determine the physiological activity depend on fluorescence-based microscopic quantification, MPN (most probable number) enumeration as well as optical density (OD_600_) value, which are commonly used to monitor cell growth (Weber et al., 2006; Klueglein and Kappler, 2013). Nevertheless, these methods can not exactly reflect the cell activity. In terms of microscopic quantification, the fluorescence dyes are probably able to penetrate the membranes of dead cells, resulting in the detection of the fluorescent signal of dead cells (Klueglein and Kappler, 2013), thus overestimating the number of live cells. MPN enumeration, which is cultivation based, is highly dependent on the culture conditions. Therefore, some viable but non-culturable or metabolically inactive cells may be omitted by the MPN enumeration owing to unsuitable growth conditions. Hence, a proper understanding of the process of ANDFO requires a reliable and efficient method to trace the real physiological state of the functional cells during Fe(II) oxidation. The nucleic acid dye PMA is a dye that can penetrate damaged cell membranes and integrate into the DNA of the cells. Since PMA-bound DNA cannot be amplified, the use of PMA allows differentiation between living and dead cells (Nocker et al., 2007; Vesper et al., 2008; Zhang et al., 2015). Another approach that can be used to discriminate between living and dead cells is the use of Raman spectroscopy combined with confocal microscopy (Berry et al., 2015; Wang et al., 2016). Lipids from organisms display a predominance of protons derived from water (or ^2^H^+^ from ^2^H_2_O). Protons can be incorporated into lipids via pathways of the known fatty acid biosynthesis (Berry et al., 2015; Wang et al., 2016). Experiments based on labeling with deuterium (D = ^2^H) have previously demonstrated that Raman spectra with signals of C-D (C-^2^H) stretching vibrations ranging from 2000 to 2300 cm^–1^ could be detected from living cells, while no signals appeared in dead cells (Berry et al., 2015; Wang et al., 2016).

In this study, we used a nitrate-dependent Fe(II)-oxidizer *B. ferrooxidans* isolated from a paddy soil (Zhou et al., 2018). It is capable of utilizing ferrous iron as electron donor with nitrate as electron acceptor under anaerobic condition. However, ANDFO by *B. ferrooxidans* ceased after 3 to 6 days of cultivation although there was still sufficient amount of ferrous iron and nitrate remaining in the medium. Furthermore, the activity of *B. ferrooxidans* decreased gradually after continuous transfers to fresh medium during ANDFO. This phenomenon was also observed for previously identified nitrate-dependent Fe(II)-oxidizers, which could not be transferred continuously in a lithoautotrophic medium (Kappler, 2005; Muehe et al., 2009). Therefore, our objective was to investigate the physiological activity of cells of *B. ferrooxidans* during ANDFO. PMA-amended 16S rRNA gene quantification combined with D-isotope based Raman spectra was utilized to differentiate the living cells from the dead ones. Scanning electron microscopy (SEM) and x-ray diffraction (XRD) were also employed to observe the morphological changes of the cells and the Fe phase transformation during Fe(II) oxidation.

## Materials and Methods

### The Microorganism and Growth Media

The strain of *B. ferrooxidans* was isolated from a paddy soil collected in Yingtan, China (116°82′E, 28°2′N) (Zhou et al., 2016, 2018). For routine cultivation of the strain, we used a mineral culture medium (pH 6.8–7.2) amended with 10 mM NaNO_3_ and 10 mM FeCl_2_. The cultures were started by adding 20 ml of a well-mixed mixture of anoxic Fe(II)-oxidizing medium (FeOM) mixed with 10% (v/v) bacterial suspension into 58 mL serum bottles. The serum bottles were then incubated in the dark at 30°C in the anaerobic chamber (N_2_ : CO_2_ : H_2_ = 90 : 5 : 5; Shel Lab Bactron IV, United States). FeOM was prepared by amending a mineral medium (see below) with vitamin solution (1 ml L^–1^) (Lovley and Phillips, 1988), trace element solution SL10 (1 ml L^–1^) (Lovley and Phillips, 1988), selenite-tungstate solution (1 ml L^–1^) (Kappler et al., 2005), bicarbonate buffer (22 mM), NaNO_3_ (10 mM) and FeCl_2_ (10 mM). The mineral medium contained MgSO_4_⋅7H_2_O (0.5 g L^–1^), CaCl_2_⋅2H_2_O (0.1 g L^–1^), NH_4_Cl (0.3 g L^–1^), and KH_2_PO_4_ (0.6 g L^–1^) (Ratering and Schnell, 2001; Amstaetter et al., 2012; Yang et al., 2015). The mineral medium was autoclaved (120°C for 20 min). FeCl_2_ was added after the mineral medium was cooled to room temperature under N_2_/CO_2_ (80/20%) (Hegler et al., 2008; Klueglein and Kappler, 2013; Klueglein et al., 2015). Then, Fe(II) carbonates and Fe(II) phosphates formed, and the precipitate was removed by sterile 0.22 μm filters after 3 days in the anaerobic glove box (Klueglein and Kappler, 2013). The final concentration of Fe(II) [dissolved Fe(II)] was calculated to be 5–8 mM. The FeOM plates were prepared by addition of agar (1.5%, w/v) in the FeOM medium.

We found that the iron oxidation capacity of the strain *B. ferrooxidans* decreased with increasing numbers of transfers, and almost no growth was observed in the FeOM. However, *B. ferrooxidans* grows well in R2A medium [yeast extract, 0.5 g; proteose peptone, 0.5 g; casamino acids, 0.5 g; glucose, 0.5 g; soluble starch, 0.5 g; K_2_HPO_4_, 0.3 g; MgSO_4_⋅7H_2_O, 0.05 g; sodium pyruvate, 0.3 g and deionized water, 1000 ml (pH 7.2)] and LB (yeast extract, 5 g; peptone, 10 g; NaCl, 10 g and deionized water, 1000 ml) medium (both of these two medium contained organic carbon) (Zhou et al., 2018). Hence, in order to keep the iron oxidation activity of the strain, we streaked it on R2A plates and incubated it at 30°C in the dark (in the anaerobic glove box) for routine cultivation (Zhou et al., 2018).

### Experimental Setup

Serum bottles (58 mL) were sterilized and dried. The empty bottles were moved to the anaerobic glove box to replace the air with gas (N_2_ : CO_2_ : H_2_ = 90 : 5 : 5) for 3 days. Then, the bottles were filled with 20 mL FeOM (pre-purged with N_2_/CO_2_) and sealed with butyl stoppers and aluminum crimps.

Two experiments were carried out in this study. Experiment 1 was performed to investigate whether the strain *B. ferrooxidans* is a nitrate-dependent Fe(II)-oxidizer, for which we prepared six treatments (*n* = 3, each) as detailed in Table 1. The bacterial colonies on the R2A plates were removed using anoxic sterile 0.9% NaCl (w/v) and harvested (8000 g, 10 min). The cells were then washed three times with anoxic distilled water and resuspended in 60 mL distilled water. The OD_600_ value of the cell suspension was 0.936 (determined with a UV/Vis spectrometer, UV3600, Shimadzu, Japan). Experiment 2 aimed to investigate the effect of initial cell concentrations on the rate of Fe(II) oxidation, and four treatments (*n* = 3, each) were set up as listed in Table 1. Cells of strain *B. ferrooxidans* were obtained as described above, and OD_600_ value of *B. ferrooxidans* suspension was 1.428.

**TABLE 1:** Description of treatments in the Experiment 1 and Experiment 2.

**Experiment**	**Treatment**	**Description**	**Incubation condition**
**Experiment 1**	Sterile (NO_3_^–^)	0.5 mL anoxic and distilled water + 20 mL FeOM without FeCl_2_ addition	
	Sterile [Fe(II)]	0.5 mL anoxic and distilled water + 20 mL FeOM without NaNO_3_ addition	
	Sterile [Fe(II) + NO_3_^–^]	0.5 mL anoxic and distilled water + 20 mL FeOM	
	*B. ferrooxidans* (NO_3_^–^)	0.5 mL cell suspension + 20 mL FeOM without FeCl_2_ addition	
	*B. ferrooxidans* [Fe(II)]	0.5 mL cell suspension + 20 mL FeOM without NaNO_3_ addition	Incubated in dark at 30 °C in the anaerobic chamber.
	*B. ferrooxidans* [Fe(II) + NO_3_^–^]	0.5 mL cell suspension + 20 mL FeOM	
**Experiment 2**	Control (Sterile)	1 mL of anoxic and distilled water + 20 mL FeOM	
	*B. ferrooxidans* (1×)	1 mL of the *B. ferrooxidans* suspension + 20 mL FeOM	
	*B. ferrooxidans* (10^–1^)	1 mL of *B. ferrooxidans* suspension (10-fold dilution) + 20 mL FeOM	
	*B. ferrooxidans* (10^–2^)	1 mL of *B. ferrooxidans* suspension (100-fold dilution) + 20 mL FeOM	

### Chemical Analyses

The concentration of ferrous iron [Fe(II)] was determined with the ferrozine assay, which was performed using sulfamic acid but not HCl for preventing oxidation of Fe(II) by the nitrite at acidic pH (Klueglein and Kappler, 2013; Zhou et al., 2019). For all setups, 900 μL of 40 mM sulfamic acid was mixed with 100 μL of culture suspension. The culture suspension was obtained from the serum bottles using a syringe in the anaerobic chamber. The mixture was incubated for 1 h at room temperature (Klueglein and Kappler, 2013). An aliquot of 20 μL of the extract was added into the 180 μL ferrozine solution (1 g ferrozine in 50 mM HEPES buffer, pH = 7). The formed ferrous complex (purple) was quantified at 562 nm using UV/Vis spectrometer. The concentrations of NO_3_^–^ and NO_2_^–^ were analyzed with ion chromatography (Dionex ICS-3000 system, Diones, Sunnyvale, CA, United States).

Iron mineralogy was examined using XRD (X’Pert PRO MPD, PANalytical B. V.) (Amstaetter et al., 2012). Ferrihydrite crystals were harvested from the samples by centrifugation (14000 *g*, 15 min) and the supernatant was removed. The crystals were washed three times with distilled water and then one time with 100% ethanol (v%) (Wu et al., 2014). After drying, the ferrihydrite crystals were ground with an agate mortar. The powder of ferrihydrite was covered by aluminum foil and packaged in air-tight bags, which were stocked in the anaerobic chamber until analysis. All of these operations were performed in the anaerobic glove box. The XRD device (operated at 40 kV, 40 mA) exhibited a broad signal in a 2θ range from 10 to 80°. The mineral phases were analyzed by X’Pert High Score Plus software equipped with PDF-database licensed by ICDD (International Centre for Diffraction Data).

### Raman Spectroscopy Analysis

For Raman analysis, we used a confocal Raman system (Horiba Jobin Yvon S. A. S, France) set up with an integrated Olympus BXFM microscope equipped with 600 g/mm grating (Cui et al., 2013). Excitation was provided by 532 nm laser with the power of 50 μW on the sample. A 100× objective lens (Olympus) was used to focus the laser beam and collect the Raman signal. Lateral and axial resolution was ca. 1 μm and ca. 2 μm, respectively. We used an acquisition time of 1 s and a Raman spectrum ranging from 500 to 2500 cm^–1^. The wavelength calibration was performed by focusing the laser beam (532 nm) on a silicon wafer with 100× objective, which presented the first-order phonon band of silicon at 520.6 cm^–1^. Usually, twenty Raman spectra were acquired from different areas chosen randomly on each sample in total.

Samples (4 mL cell suspension) were taken from cultures of *B. ferrooxidans* (1×) after 120 h incubation (around the logarithmic phase) in the anaerobic chamber. The suspension was divided into two parts (each part was 2 mL solution in average). In order to investigate the metabolic activity of cells during ANDFO, the first part was mixed with 2 mL anoxic D_2_O (D-99.9% atom %; Sigma-Aldrich) (*B. ferrooxidans* + FeOM + D_2_O) directly and incubated in the dark at 30°C in the anaerobic chamber. The other part was mixed with 2 mL LB liquid medium and 4 mL anoxic D_2_O (*B. ferrooxidans* + LB + D_2_O) and incubated under the same conditions, which aimed at further exploring whether cells were in the state of metabolic inactivity or death. An aliquot of 2 μL of the mixture was dropped onto the glass slide plated with gold on the surface and dried on the super clean workbench for 30 min for Raman spectroscopy analysis after 1, 2, 5, 16, 24, and 48 h incubation (Cui et al., 2013).

### Scanning Electron Microscopy

Colonies grown on the R2A plates and FeOM were harvested and the bacterial suspension was fixed with 2.5% (w/v) glutaraldehyde for 2 h and centrifuged at 6000 *g* for 15 min. Cells were dehydrated in a graded series (30, 50, 70, 90, and 100%; v/v) of ethanol solutions for 10 min each. The cells were harvested by centrifugation (6000 *g*, 15 min) and re-suspended in 100% ethanol. The suspension was dropped onto the aluminum foil and covered with filter paper, which was dried by critical point drying. Aluminum foil (attached with bacteria) was adhered to the conductive tapes (spi, America) mounted on copper stubs and coated with gold. The SEM (S4800, Hitachi) device was operated at 5 kV and 6 mm working distance with a secondary electron detector.

### PMA-Amended Quantitative PCR

Samples of bacterial cells [experiment 2, *B. ferrooxidans* (1×)] were harvested by centrifugation (14000 *g*, 15 min) on 0, 12, 24, 48, 96, and 120 h, respectively. Propidium monoazide (PMA; Biotium, Inc., Hayward, CA, United States) was prepared before DNA extraction. One milligram PMA was re-suspended in 65.4 μL of dimethyl sulfoxide (DMSO) (resulting in a final concentration of 34.6 mM) and stocked in the dark at −20°C until use (Vesper et al., 2008). Precipitates were re-suspended in 2 mL of 0.1 mM sterile PBS. After vortex, the mixtures were supplemented with 2 μL PMA stock solution (final concentration of PMA was ca. 30 μM) and incubated for 5 min with occasional mixing in the dark at room temperature (Zhang et al., 2015). Then, the samples were exposed to a 650 W halogen light source (220V, 3400K, OSRAM, Munich, Germany) for 15 min. To avoid excessive heating, the sample tubes were horizontally laid on ice, and kept on the top of the ice 18 cm from the light source. Cells were then pelleted at 14000 *g* for 10 min after photo-induced cross-linking. The precipitates were washed with 2 mL PBS (0.1 mM) prior to DNA extraction [using FastDNA Spin Kit (MP Biomedical, France)] according to the manufacturer’s protocol.

Real-time PCR Detection System (Roche 480, Roche, Indianapolis, IN, United States) was utilized to assess the abundance of the bacterial 16S rRNA genes. The aim was to investigate the dynamic number of living *B. ferrooxidans* cells during the incubation. The amplification was performed in triplicates using the primer set of 515F-907R for the bacterial 16S rRNA gene (Stubner, 2002). The thermal cycling conditions are detailed in Supplementary Table S1. The reaction mixture contained 2 μL DNA as a template, 0.8 μL of each primer, 10 μL of SYBR 2 Premix EX Taq, 0.6 μL of BSA (20 mg mL^–1^), and 5.8 μL of ddH_2_O and the reaction contained no DNA template in the negative control. Soil (Yingtan, China) sample DNA was used to clone these genes to prepare standard plasmids. Standard curves were generated using 10-fold serial dilutions of the standard plasmids (Yang et al., 2015). We detected only one peak at the melting temperature (Tm) of 82.5°C indicating the specificity of amplicons. Only reactions with efficiencies ranging from 90 to 110% were accepted (Yang et al., 2015).

### Statistical Analyses

Statistical tests, including analysis of variance (ANOVA) and Pearson correlation analysis, were performed by SPSS 18.0 (SPSS Inc., Chicago, IL, United States) and Origin 9.0 (Inc., OriginLab, United States). Statistical significance was performed by Duncan’s multiple range test and denoted at *P* < 0.05.

## Results

### Iron(II) Oxidation and Nitrate Reduction of the Strain *B. ferrooxidans* in the FeOM

**FIGURE 1:**
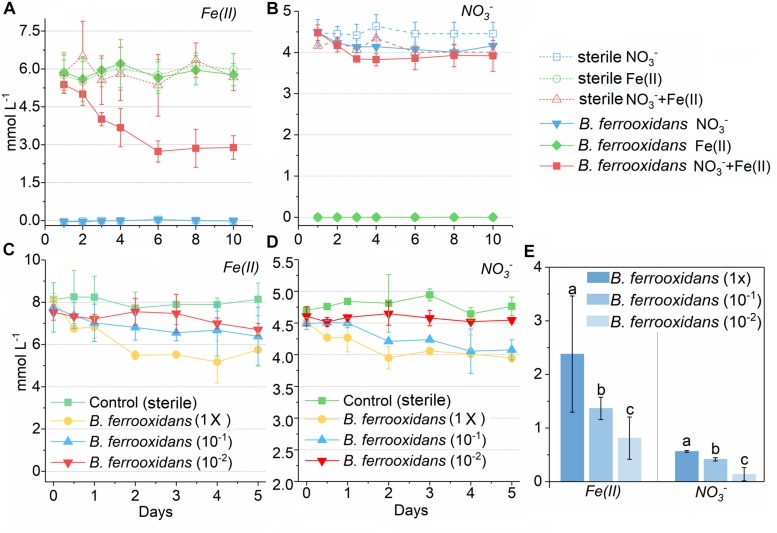
Concentration kinetics of ferrous iron and nitrate in two experiment during incubation. Concentration kinetics of ferrous iron **(A)** and nitrate **(B)** in the media containing NO_3_^–^, Fe(III) as well as both NO_3_^–^ and Fe(III) during 10 days incubation with or without presence of the bacterium *Bacillus ferrooxidans*, respectively. Concentration kinetics of ferrous iron **(C)** and nitrate **(D)** in the FeOM during 120 h incubation with different concentrations of *B. ferrooxidans* cells. The error bars represent standard deviations of three replications. FeOM represented the FeOM. **(E)** Total amount of Fe(II) and NO_3_^–^ consumed in treatments of *B. ferrooxidans* (1×), *B. ferrooxidans* (10^–1^), and *B. ferrooxidans* (10^–2^) during the incubation. Different letters (a–c) in the bar graphic indicate significant difference between the treatments at *P* < 0.05.

No iron(II) oxidation was observed in the medium without NO_3_^–^ and no nitrate reduction was detected in the medium without Fe(II) addition as well during 10 days of incubation (Figures 1A,B). In comparison, iron(II) and NO_3_^–^ were consumed in the FeOM incubated with cells of *B. ferrooxidans*. The concentrations decreased by 2.49 and 0.56 mM, respectively, and the consumption almost ceased after 120-h incubation (Figures 1A,B).

To determine the effect of different cell concentrations on the iron(II) oxidation, the FeOM was inoculated with cells with final concentrations of 3.2 × 10^6^ cells mL^–1^ [*B. ferrooxidans* (1×)], 3.2 × 10^5^ cells mL^–1^ [*B. ferrooxidans* (10^–1^)], and 3.2 × 10^4^ cells mL^–1^ [*B. ferrooxidans* (10^–2^)], respectively. After a 120-h incubation, this dilution sequence exhibited a concentration-dependent effect on the iron(II) oxidation activity compared with the abiotic setup (Figures 1C,D). The treatment of *B. ferrooxidans* (1×) showed significantly (*P* < 0.05) higher extent in Fe(II) oxidation (2.38 ± 0.16 mM) than that in the treatments of *B. ferrooxidans* (10^–1^) (1.36 ± 0.21 mM) and *B. ferrooxidans* (10^–2^) (0.64 ± 0.11 mM) (Figures 1C,E). Similarly, the extent of nitrate reduction was highest in the setup of *B. ferrooxidans* (1×) among three setups (Figures 1D,E). The dynamic of Fe(II) oxidation in the setup of *B. ferrooxidans* (1×) showed that the iron(II) oxidation approximately stopped after 48-h incubation.

### Colonies of Strain *B. ferroxidans* Grown on Fe(II)-Oxidizing Agar Plates and R2A Plates

Colonies of strain *B. ferroxidans* grown on Fe(II)-oxidizing agar plates showed significant iron(II) oxidation during incubation (Supplementary Figure S1). However, no proliferation was observed when these colonies were transferred to fresh Fe(II)-oxidizing plates (data not shown). To further investigate the physiological activity, these colonies were further transferred to fresh R2A plates and new colonies were formed after a 120-h incubation under anaerobic conditions (Supplementary Figure S1). Moreover, the colonies on the R2A plates still possessed the capability of ANDFO (Supplementary Figure S2).

### Quantification of Cells of the Strain *B. ferrooxidans* in the FeOM

**FIGURE 2:**
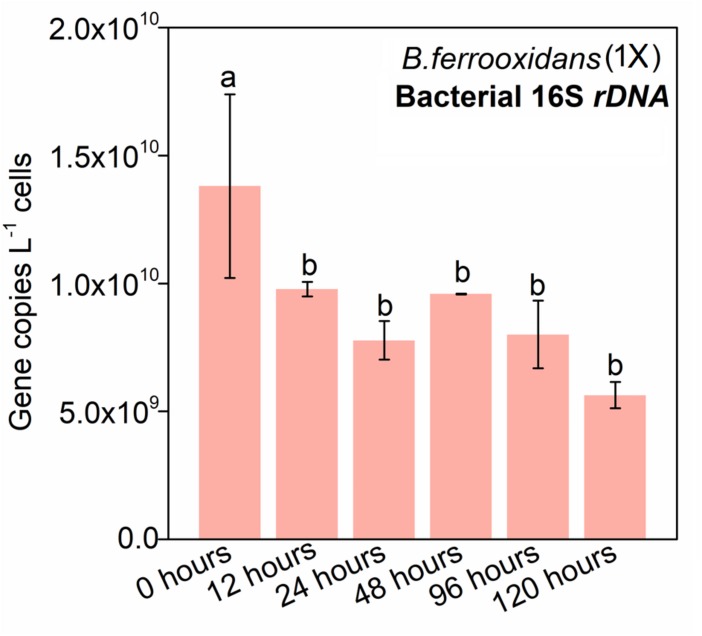
Gene copy numbers of bacterial 16S *rDNA* in the treatment of *B. ferrooxidans* (1×) during the 120-h incubation. Pink bars represented the 16S *rRNA* copy numbers in the treatment of *B. ferrooxidans* (1×) amended with PMA over time. The error bars represent standard deviations of three replications. Statistical significance was performed by Duncan’s multiple range test and denoted at “a” and “b.”

Quantitative PCR of the 16S rRNA gene was used to investigate the dynamic of the living cells. The abundance of 16S rRNA gene at 0 h was 3.41 × 10^10^ ± 7.55 × 10^8^ copies L^–1^ cells, which was 4.03 × 10^9^ copies L^–1^ cells higher than that at 12 h (Figure 2). However, there was no significant difference in the dynamic of the 16S rRNA abundances with PMA-amended DNA as templates in the setup of *B. ferrooxidans* (1×) during the following 108-h incubation (Figure 2).

### Raman Spectroscopy of the Strain *B. ferrooxidans*

**FIGURE 3:**
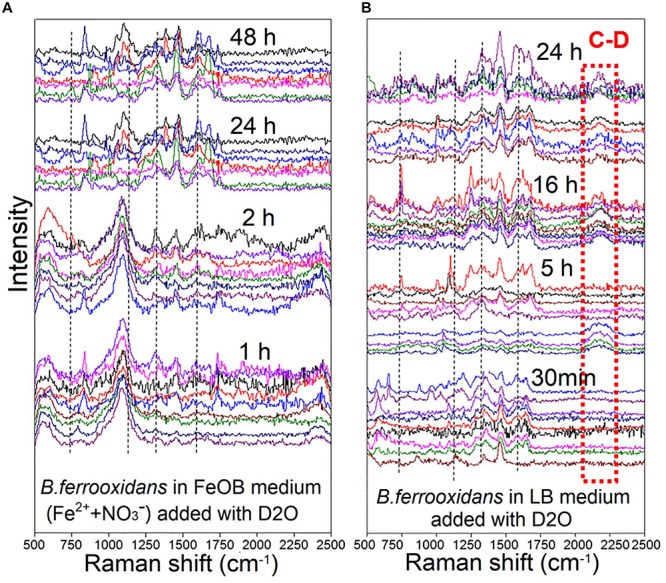
Raman spectra of *B. ferrooxidans* cells cultured in the treatments [*B. ferrooxidans* + FeOM + D_2_O **(A)** and *B. ferrooxidans* + LB + D_2_O **(B)**]. FeOM represented the Fe(II)-oxidizing medium.

For the setup *B. ferrooxidans* (1×) after the 120-h incubation, Raman spectra of cells cultivated in FeOM supplemented with heavy water (D_2_O; 50%; v/v) without/with LB medium showed many intense Raman bands (Figure 3). Four characteristic bands of cytochrome c (741, 1121, 1311, and 1587 cm^–1^) were observed in the two setups (Figure 3). In the setup of *B. ferrooxidans* + FeOM + D_2_O, there was no detectable peaks in the region between 2040 and 2300 cm^–1^ after 1, 2, 24, and 48 h incubation (Figure 3A). In contrast, a detectable peak in the same region appeared after a 5-h incubation in the treatment of *B. ferrooxidans* + LB + D_2_O (Figure 3B). Based on the spectra, it was calculated that none of the cells in the treatment of *B. ferrooxidans* + FeOM + D_2_O contained the broad peak during the 48-h incubation (Table 2). For the treatment of *B. ferrooxidans* + LB + D_2_O, the ratio of cells containing the broad peak increased with incubation time and the majority of cells contained the peak after 16–48 h incubation (Table 2).

**TABLE 2:** The ratio of C-D of Raman spectra detected from the cell incubation in treatments of *B. ferrooxidans* + FeOM + D_2_O and *B. ferrooxidans* + LB + D_2_O, respectively. FeOM represented the Fe(II)-oxidizing medium.

**Incubation time**	**Ratio of C-D (%)**	
	***B. ferrooxidans* + FeOM + D_2_O**	***B. ferrooxidans* + LB + D_2_O**
30 min	0	0
1 h	0	0
2 h	0	0
5 h	0	26.09%
16 h	0	94.44%
24 h	0	100%
48 h	0	97.22%

### SEM Observation and Mineral Formation

**FIGURE 4:**
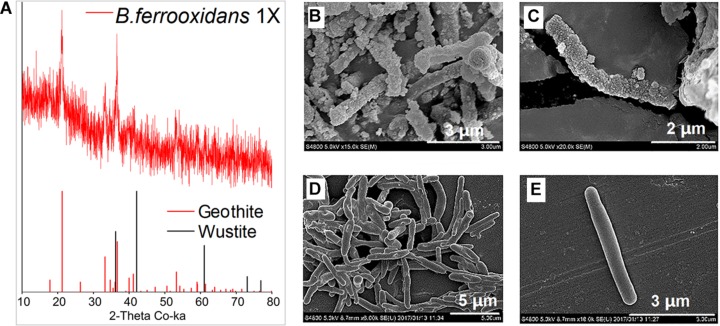
XRD analysis of minerals formed by microbial oxidation **(A)** and SEM images of *B. ferrooxidans* cultured in the FeOM **(B,C)** and R2A medium **(D,E)**.

X-ray diffraction analysis showed the role of biotic Fe(II) oxidation on mineral formation (Figure 4A). After 120-h incubation, goethite was observed in the FeOM (Figure 4A).

Scanning electron microscopy images revealed that the cells were heavily encrusted with particles when cultivated in FeOM (Figures 4B,C), while no encrustations were observed on cells growing in the R2A medium (Figures 4D,E).

## Discussion

### Nitrate-Dependent Fe(II)-Oxidation by Strain *B. ferrooxidans*

Periodic changes of paddy soil generate an anoxic environment after flooding (Ratering and Schnell, 2001) and ANDFO is known to occur in soil where oxygen is depleted (Ratering and Schnell, 2001; Weber et al., 2006). *B. ferrooxidans* was isolated from a paddy soil and found to be capable of anaerobic iron(II) oxidation (Figure 1 and Supplementary Figures S2, S3). No Fe(II) oxidation was observed in the FeOM without cell addition as well as the medium free of nitrate (Figure 1 and Supplementary Figures S2, S3), suggesting that the strain is a nitrate-dependent Fe(II)-oxidizing bacterium. In addition, no significant change of ammonium concentration was observed in treatments inoculated with cells during the incubation (data not shown). We calculated that the molar ratio of Fe(II)-oxidized to nitrate reduced (ca. 5:1, Figure 1E) by the strain *B. ferrooxidans* was consistent with the theoretical stoichiometry (5FeCO_3_ + NO_3_^–^ + 12H_2_O → 5Fe(OH)_3_ + 0.5N_2_ + 4H^+^ + 5HCO_3_^–^; 4FeCO_3_ + NO_3_^–^ + 9.5H_2_O → 4Fe(OH)_3_ + 0.5N_2_O + 3H^+^ + 4HCO_3_^–^) (Weber et al., 2006; Melton et al., 2014). Nitrite could not be detected after 24 h (only detected during 12 to 24 h, data not shown), meanwhile N_2_ (0.12 mM), and N_2_O (0.14 mM) were detected after ANDFO in treatment of *B. ferroxidans* (Supplementary Table S2). It was suggested that NO_2_^–^, N_2_ and N_2_O were end products during the ANDFO. Owing to the pre-cultivation, nitrate reduction in cells of strain *B. ferroxidans* may also couple to oxidation of dead biomass or organic carbons stored from R2A medium during pre-incubation, which supported the fact that nitrate concentration decreased in the treatment of *B. ferrooxidans* (NO_3_^–^) (Figure 1B). The product nitrite would serve as oxidant resulting in abiotic iron(II) oxidation (NO_2_^–^ + H^+^ ⇌ HNO_2_; 2HNO_2_ →NO_2_ + NO + H_2_O; NO_2_ + 2Fe^2+^ + 2H^+^ →2Fe^3+^ + NO + H_2_O; NO + Fe^2+^ + H^+^ →Fe^3+^ + HNO; 2HNO →N_2_O + H_2_O) (Klueglein and Kappler, 2013). During ANDFO, only inorganic carbon (NaHCO_3_) was amended in the FeOM, and NaH^13^CO_3_-labeled incubation experiment displayed significant capability for CO_2_ assimilation of *B. ferrooxidans* during ANDFO (Supplementary Figure S5 and Supplementary Table S3). Our previous study demonstrates that this strain is able to utilize a variety of organic substrates followed by production of acids (Zhou et al., 2018). All of these suggested a mixotrophic growth of strain *B. ferroxidans.* Interestingly, the addition of acetate (5 mM) in the FeOM did not result in a significantly higher extent of Fe(II) oxidation when compared to the setup without acetate addition (Supplementary Figure S3, S4). Further studies are needed to further explore the underlying mechanism.

### The Metabolic Inactive State of *B. ferrooxidans* Cells During ANDFO and Re-awakening

The strain of *B. ferrooxidans* showed incomplete ability of ANDFO during the 120-h incubation (Figure 1). Similarly, the mixotrophic *Acidovorax* sp. strain 2AN showed incomplete ANDFO when the medium was free of organic substrates (Chakraborty et al., 2011). For this strain, iron oxidation ceased even though there was still a large amount of nitrate and Fe(II) available in the medium, with only 29.2% of total Fe(II) and 12.6% of NO_3_^–^ consumed in the setup of *B. ferrooxidans* (1×) (Figure 1). Additionally, the activity of Fe(II) oxidation decreased with successive transfers, and almost no Fe(II) oxidation was observed in the third generation (data not shown). These results indicate that the Fe(II) oxidation might link to consumption of stored organic carbons and lead to cell death or a metabolically inactive state during incubation in the FeOM.

Previous studies have indicated that PMA-amended PCR is a reliable tool to differentiate living cells from dead ones (Nocker et al., 2007; Vesper et al., 2008; Zhang et al., 2015). Limited detection of the 16S rRNA gene from fragmented cells of *B. ferrooxidans* incubated with PMA confirms that the approach with PMA amendment is robust in discriminating the dead cells from the living ones (Supplementary Figure S6). The experiment with addition of PMA showed an initial decrease in the number of living cells from 0 to 12 h, while no significant difference in the abundance of cells was observed during the remaining 108 h of incubation, which indicated that the majority of the cells had intact membranes and most cells were still alive in the FeOM (Figure 2). These results are in agreement with the previous study on the strain 2002 (Weber et al., 2006; Zhao et al., 2013), which is an anaerobic nitrate-dependent Fe(II)-oxidizing bacterium.

To further examine the physiological activities of cells after 120-h Fe(II) oxidation, D-tracing was utilized and detected via Raman spectra system. Raman spectra of *B. ferrooxidans* harvested from cultures without an organic substrate (the treatment of *B. ferrooxidans* + FeOM + D_2_O) showed no shift during 0–48 h incubation (Figure 3 and Table 2). In contrast, we observed several distinctive shifts in the treatment with organic substrate (*B. ferrooxidans* + LB + D_2_O) after 5-h incubation. This spectral pattern reflects a substitution of C-D in newly synthesized lipids in the presence of heavy water. All of these results suggested that cells were still alive and maintained a viable, but physiologically inactive state after ANDFO during the incubation, and they might revive once the environment becomes suitable. “Dead” colonies of the strain *B. ferroxidans* survived in R2A plates and then had the ability to oxidize Fe(II), which further verified our speculation. These results were in accordance with the quantification of 16S *rRNA* gene, which also indicated that cells had experienced a metabolically inactive state after Fe(II) oxidation.

### The Effect of Biotic Fe(II) Oxidation on Mineral Transformation and Cell Metabolism

The formation of goethite indicates the effect of the isolated strain *B. ferrooxidans* on iron cycling in the environment (Figure 4A). This is comparable to the mineral transformation during ANDFO by *Acidovorax* sp. (BoFeN1), which formed green rust as an intermediate phase at the initial stage and goethite after complete Fe(II) oxidation in the comparable growth medium (Pantke et al., 2012; Klueglein et al., 2014). As for the ANDFO, it was likely that nitrate reduction was coupled to oxidation of organics stored inside of cells during pre-cultivation in the R2A medium. However, no additional organic substrates were added to the FeOM. Therefore, ADNFO involved consumption of pre-stored organic matters may be responsible for the induction of a metabolically inactive state in *B. ferrooxidans*. Heavy cell encrustations formed during ANDFO is a common characteristic of nitrate-dependent Fe(II)-oxidizers (Kappler et al., 2005; Chakraborty et al., 2011; Klueglein and Kappler, 2013), and the encrustations would probably hinder nutrient uptake and metabolite efflux (Chakraborty et al., 2011; Klueglein et al., 2014). Cell encrustations has been suggested as another potential factor leading to slower cell metabolism (Coby and Picardal, 2005; Klueglein et al., 2014). In addition, Superoxide dismutase activity of cells significantly increased after ANDFO (Supplementary Table S4), which suggested that toxicity of aqueous Fe(II) (5∼8 mM in our study) in the mM range could also have negative impact on physiological activity of cells (Coby and Picardal, 2005; Poulain and Newman, 2009; Bird et al., 2013; Klueglein et al., 2015). Significant amount of exopolysaccharides surrounding and agglutinating the cells has been identified in various strains, such as *Acidovorax* sp. (BoFeN1) and the purple phototrophic bacterium *Rhodopseudomonas palustris* TIE-1, which might be a response to the toxic effect on the cells (Bird et al., 2013; Klueglein et al., 2014, 2015).

### The Potential Inhibition of Iron(II) Oxidation on Further Turnover of Nitrogen in the Environment

Previous studies have demonstrated that ANDFO is widespread among a variety of bacteria in marine, soil, wetland, and freshwater (Straub and Buchholzcleven, 1998; Weiss et al., 2003; Kappler, 2005). The process of ANDFO links closely to denitrification, and most mixotrophic nitrate-dependent Fe(II)-oxidizing bacteria are actual denitrifiers to some extent (Klueglein and Kappler, 2013). Our strain belongs to the genus of *Bacillus*, in which numbers of members possess the common feature of denitrification (Boone et al., 1995; Weber et al., 2006; Verbaendert et al., 2011). Moreover, PCR with specific primers demonstrated that *B. ferrooxidans* harbored denitrifying genes, including *narG* (420 bp), *nasA* (756 bp), and *nosZ* (435 bp) (Supplementary Table S1), which probably played a key role in nitrate reduction during ANDFO (Kandeler et al., 2006; Ligi et al., 2014). However, it is well documented that continuous cultivation of nitrate-dependent Fe(II)-oxidizers is impossible (Kappler, 2005; Muehe et al., 2009), probably because these organisms enter into metabolic dormancy during ANDFO. Further nitrate reduction would stop in the metabolically inactive cells after ANDFO, and chemical Fe(II) oxidation by NO_2_^–^ would hence cease. Therefore, it is likely that complete denitrification would be inhibited in denitrifiers after the ANDFO process in the environments. The previous study on the denitrifying strain *Shewanella putrefaciens* 200 show that NO_X_^–^ reduction was inhibited in the presence of a variety of Fe(III) oxides, including hematite, goethite and iron-bearing natural sediments (Coby and Picardal, 2005). Fe(II)-oxidizing bacteria (including acidophilic, aerobic iron oxidizers; neutrophilic, aerobic iron oxidizers; neutrophilic, anaerobic (nitrate-dependent) iron oxidizers; and anaerobic photosynthetic iron oxidizers) are abundant in wetland ecosystems and even dominant in the rhizosphere microbial communities and lead to formation of goethite, hematite (Weiss et al., 2003; Hedrich et al., 2011), which suggests a potentially negative impact of iron(II) oxidation on nitrogen cycling in the environments.

Our study suggests that the failure of continuous cultivation of the reported Fe(II)-oxidizers may be due to induction of a metabolically inactive state of cells during iron(II) oxidation. These cells could be awakened and enter into the normal metabolic state under suitable conditions. Many denitrifiers are facultative anaerobes and they gain energy from oxygen to rapidly grow and reproduce (Gottschal and Szewzyk, 1985). For the ANDFO-induced metabolically inactive denitrifiers in paddy soils and wetlands, alternate wetting and drying conditions may give them new life during drainage and prepare them for the next round of denitrification under flooded conditions. Hence, our results emphasize the problems with DNA-level based functional gene quantification as an important approach to determine the abundance of microorganisms with different functional attributes. These methods may seriously overestimate related microbial activity, including denitrification especially in this ferric iron-rich zone, including rhizosphere of most wetland and submersed aquatic plants.

In summary, the novel ANDFO bacterium, *B. ferrooxidans*, isolated from a paddy soil, showed incomplete iron(II) oxidation under anaerobic conditions. ANDFO could lead to Fe mineral transformation and cell morphology change in the medium. Cell encrustation formation and Fe(II) toxicity might be the direct cause of the metabolically inactive state of *B. ferrooxidans* after Fe(II) oxidation. Our findings demonstrate that this bacterium with the ability to perform ANDFO enters into an inactive state after a few days in an inorganic medium. We suggest that a possible reason for this inactivity is that the cells get encrusted with Fe(III) minerals that hinders diffusion of substrates into the cell. Our findings indicate that ANDFO microorganisms make contribution to iron and nitrogen cycling, which linked closely to the process of iron(III) reduction and then drove the iron cycle coupled to organic matters oxidation. However, the potential role of denitrifiers in iron and nitrogen cycling might be overestimated under some anaerobic environments rich in Fe^2+^ and NO_3_^–^ but not organic substrates, since some ANDFO bacteria could enter a metabolically inactive state after iron oxidation. However, the dynamic change of organic carbon might lead to the periodic “dormancy” and “re-awakening” state of ANDFO microbes, which resulted in the constant cycling of carbon, nitrogen, and iron in natural environments.

## Author Contributions

G-WZ and X-RY designed the experimental protocol and carried out the experiments. X-RY, J-QS, LC, and B-XZ gave assistance in experiments. G-WZ wrote the manuscript. X-RY, RR, J-QS, and Y-GZ revised the manuscript. All authors read and approved the final version of the manuscript.

## Conflict of Interest Statement

The authors declare that the research was conducted in the absence of any commercial or financial relationships that could be construed as a potential conflict of interest.
