# Supplementary material for: Metabolic Inactivity and Re-awakening of a Nitrate Reduction Dependent Iron(II)-Oxidizing Bacterium Bacillus ferrooxidans
Source: Front Microbiol. 2019 Jul 3;10:1494. doi: 10.3389/fmicb.2019.01494 (PMC6617468; doi:10.3389/fmicb.2019.01494)
Supplement: Supplementary file 1 [file Data_Sheet_1.docx]

***Supplementary Material***

**Metabolic inactivity and re-awakening of a nitrate reduction dependent iron(II)-oxidizing bacterium *Bacillus ferrooxidans***

Guo-Wei Zhou^1,2^, Xiao-Ru Yang^1,^*, Regin Rønn^3^, Jian-Qiang Su^1^, Li Cui^1^, Bang-Xiao Zheng^4^, and Yong-Guan Zhu^1,2,5^

*^1^Key Lab of Urban Environment and Health, Institute of Urban Environment, Chinese Academy of Sciences, Xiamen, China*

*^2^State Key Lab of Urban and Regional Ecology, Research Center for Eco-Environmental Sciences, Chinese Academy of Sciences, Beijing, China*

*^3^Department of Biology, University of Copenhagen, Copenhagen Ø, Denmark*

*^4^Falculty of Biological and Environmental Sciences, University of Helsinki, Lahti, Finland,*

*^5^College of Advanced Agricultural Sciences, University of Chinese Academy of Sciences, Beijing, China.*

* Corresponding Author

Phone: (+86) 592 6190997; Fax: (+86) 592 6190977;

E-mail: xryang@iue.ac.cn

Number of figures: 6.

Number of tables: 4.

1. **Supplementary materials and methods**

**PMA incubation experiments.**

In order to investigate the role of PMA in distinguishing the dead cells from living cells, five experimental treatments were set, including *Bacillus ferrooxidans* PMA addition, *B. ferrooxidans* no PMA addition, *B. ferrooxidans* (cell fragment) PMA addition, *Enterobacter* sp. (TCD1-1) PMA addition, and TCD1-1 no PMA addition (TCD1-1 was a strain isolated from a paddy soil in our lab and belonged to the genus *Enterobacter*).

For *B. ferrooxidans* PMA addition and TCD1-1 PMA addition, before PMA incubation, 650 μl (*B. ferrooxidans*; OD = 0.950) and 700 μl (TCD1-1; OD = 0.723) of cell suspension cultivated overnight (30 °C, R2A culture) were incubated with PMA (final concentration of PMA was 30 µmol L^-1^ in the mixture), respectively. Then, the following operation was consistent with the method describe previously.

*B. ferrooxidans* no PMA addition and TCD1-1 no PMA addition represented the treatments without PMA incubation. 650 μl (*B. ferrooxidans*; OD = 0.950) and 700 μl (TCD1-1; OD = 0.723) of cell suspension were collected by centrifuging (14000 g, 10 min), respectively.

For *B. ferrooxidans* (cell fragment) PMA addition, 650 μl (*B. ferrooxidans*; OD = 1.350) of cell was fragmented using homogenous cruder (6.0 m s^-1^, 40 s; FastPrep®-24, MP, America), and the cell suspension was incubated with PMA (final concentration of PMA was 30 µmol L^-1^ in the mixture) as described above.

**Measurement of ^13^CO_2_ assimilation in cells**

Strain *B. ferrooxidans* was cultivated according to the method described before with replacing NaH^12^CO_3_ with NaH^13^CO_3_ (^13^C 99%, [Cambridge Isotope Laboratories](https://www.isotope.com/)) during the incubation. After 120-h incubation, cells of strain *B. ferrooxidans* in NaH^12^CO_3_ and NaH^13^CO_3_ added FeOB media were harvested (8000 g, 10 min), washed three times with anoxic distilled water, then air-dried cells were used for Raman spectroscopy and freeze-dried samples were used for measurement of ^13^CO_2_ assimilation by GC-isotope ratio mass spectrometry (Thermo Finnigan Delta V Advantage, Bremen, Germany) ([Conrad et al. 2000](#_ENREF_2); [Zhou et al. 2017](#_ENREF_7)).

**Measurement of N_2_O, N_2_, NO_2_^-^, NH_4_^+^ and SOD activity**

Headspace N_2_O and N_2_ were determined by gas chromatography with a robotized incubation system (Agilent 7890, Santa Clara, CA, US) ([Molstad et al. 2007](#_ENREF_5)). A aliquot of 1 mL liquid sample was collected for analysis of NO_2_^-^ and NH_4_^+^ in the anaerobic box. After filtrated through 0.22 μm filter, the concentrations were detected by chromatography (Dionex ICS-3000 system, Diones, Sunnyvale, CA, USA) ([Zhou et al. 2019](#_ENREF_8)). Cells were collected from R2A and FeOM after 12 hours of cultivation at 30 °C in the anaerobic, and SOD activity were analyzed using the xanthine–xanthine oxidase-nitroblue tetrazolium method ([Miyatake and Iwabuchi 2005](#_ENREF_4)).

1. **Supplementary Figures and Tables**
   1. **Supplementary Figures**


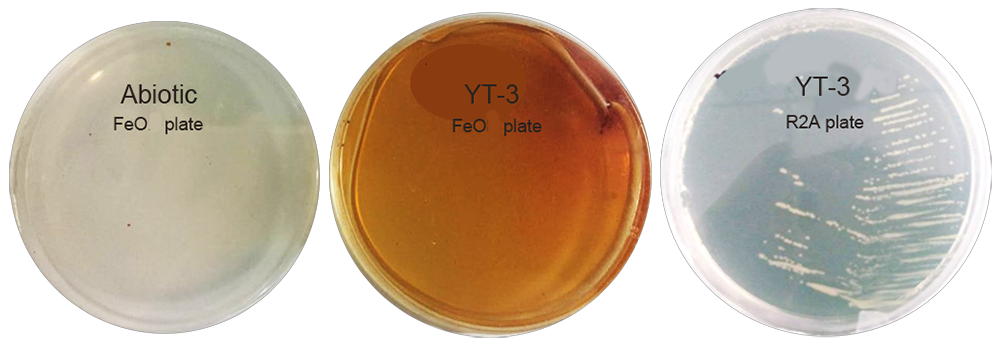
**Figure S1.** Images of *B. ferrooxidans* (YT-3) cultured on the Fe(II)-oxidizing and R2A plates at 30 °C in the anaerobic box. The “Abiotic FeO plate” represented the Fe(II)-oxidizing agar plate and was used as a control setup on which no cells were inoculated.


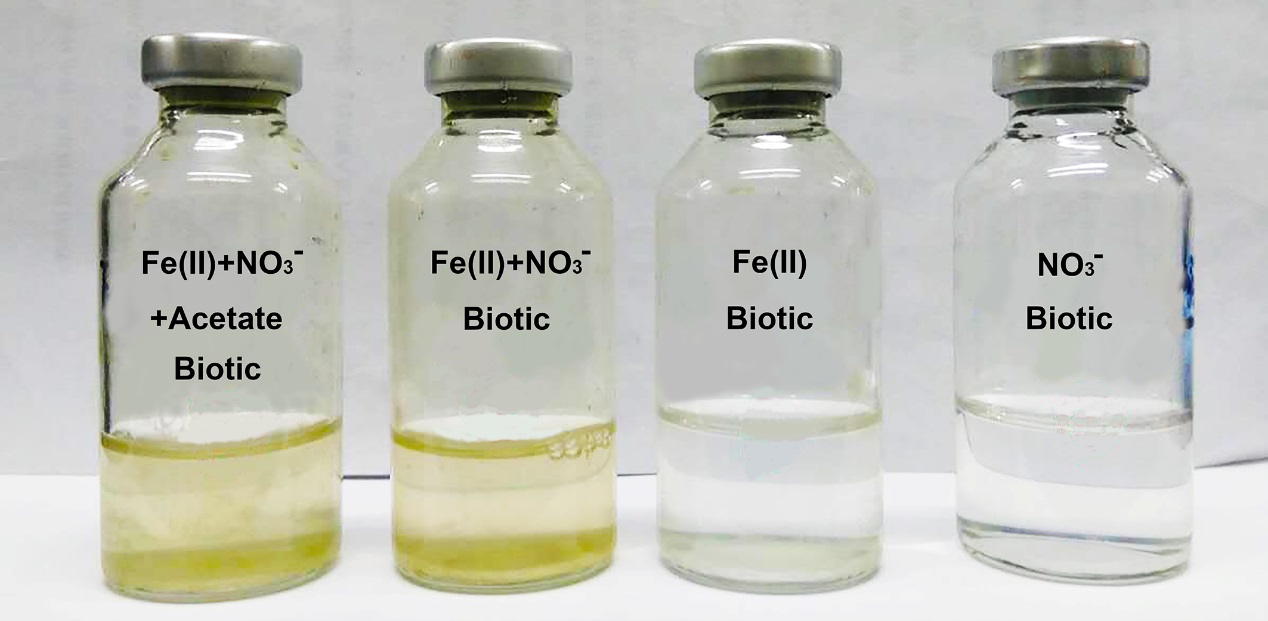


**Figure S2.** Images of *B. ferrooxidans* cultured in the different medium at 30 °C in the anaerobic box. Fe(II) + NO_3_^-^ + Acetate represented the medium contained ferrous iron, nitrate and acetate. Fe(II) + NO_3_^-^ represented the medium contained ferrous iron and nitrate. Fe(II) represented the medium contained ferrous iron. NO_3_^-^ represented the medium contained NaNO_3_.


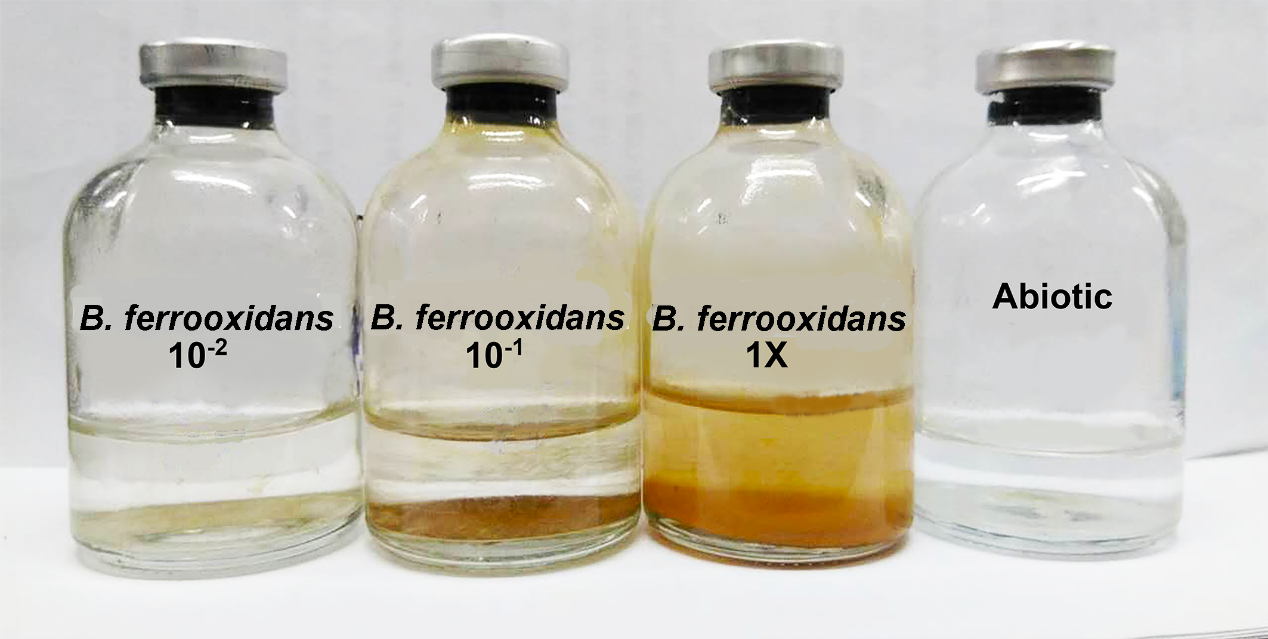


**Figure S3.** Images of *B. ferrooxidans* cultured in the Fe(II)-oxidizing medium inoculated with different densities of cells at 30 °C in the anaerobic box.


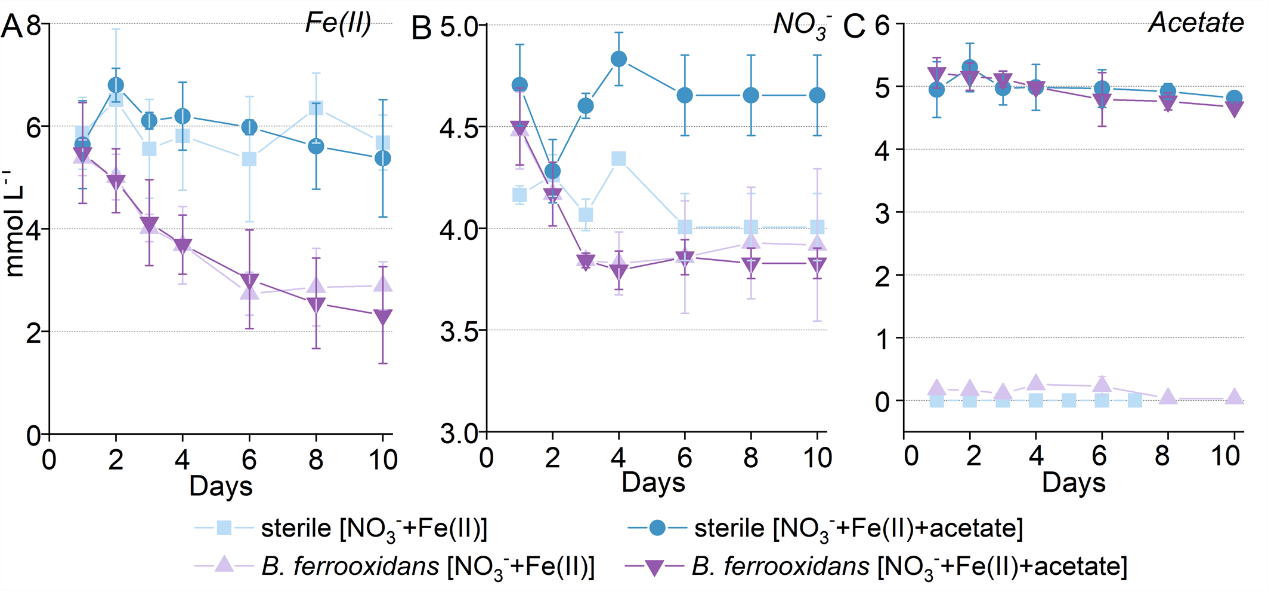
**Figure S4.** Kinetics of ferrous iron (A), nitrate reduction (B) and acetate (C) in the incubation of different medium. Fe(II) + NO_3_^-^ + Acetate represented the medium contained ferrous iron, nitrate and acetate. Fe(II) + NO_3_^-^ represented the medium contained ferrous iron and nitrate. The error bars represent standard deviations of three replications.


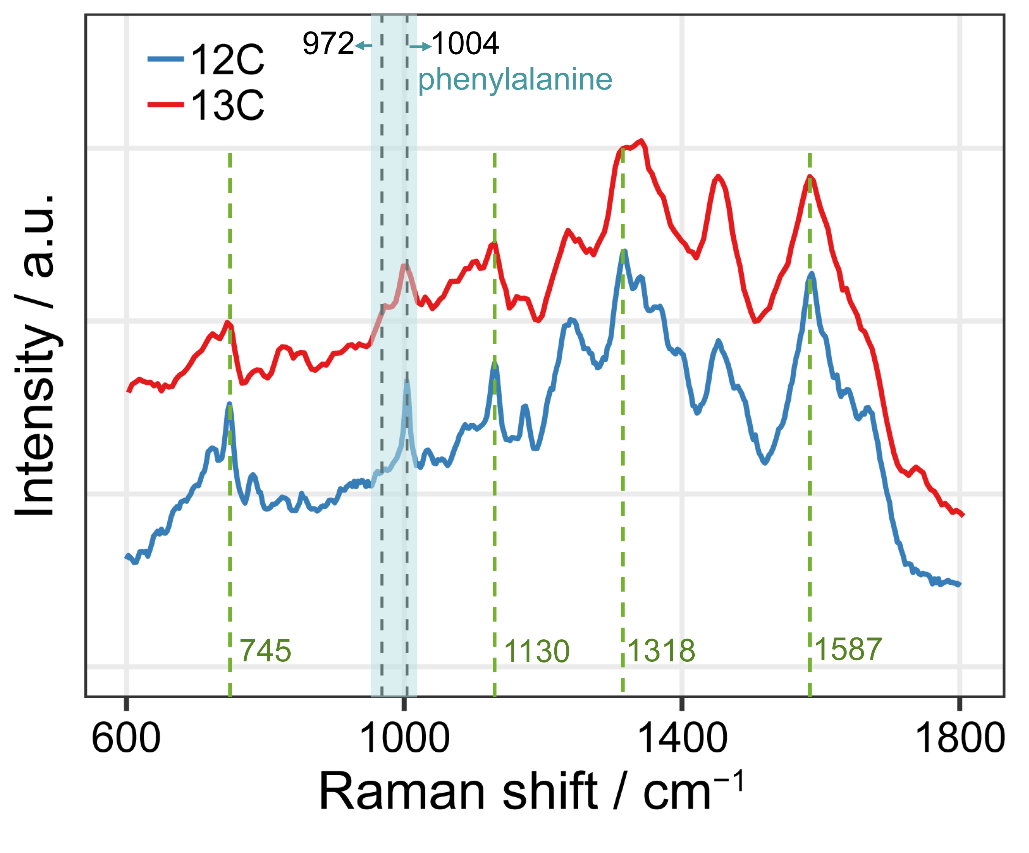
**Figure S5.** Raman spectra of *B. ferrooxidans* cells cultured in FeOB media added with NaH^12^CO_3_ (12C) and NaH^13^CO_3_ (13C). 745, 1130, 1318 and 1587 cm^-1^ were characteristic bands of cytochrome c. The shade of light blue represented the characteristic bands of phenylalanine. Shift of band from 1004 cm^-1^ to 972 cm^-1^ was observed in these two setups.





**Figure S6.** Gene copy numbers of bacterial 16S *rRNA* in six setups (*B. ferrooxidans* PMA addition, *B. ferrooxidans* no PMA addition, *B. ferrooxidans* (cell fragment) PMA addition, TCD1-1 PMA addition and TCD1-1 no PMA addition).

1. **Supplementary Tables**

**Table S1.** Primers and qPCR processes used in this study.

| Genes | Primers  (sequences 5’-3’) | | qPCR processes | References | length |
| --- | --- | --- | --- | --- | --- |
| *narG* | *NarG1960f* | TAYGTSGGSCARGARAA | 5 min at 95°C, followed by 40 cycles of 30 s at 95°C, 40 s at 58°C and 40 s at 72°C. | ([Zhang et al. 2014](#_ENREF_6)) | 420 bp |
|  | *NarG2650r* | TTYTCRTACCABGTAGC |  |  |  |
| *nasA* | nasA964  nasA1735 | CARCCNAAYGCNATGGG  ATNGTRTGCCAYTGRTC | 5 min at 95°C, followed by 40 cycles of 30 s at 95°C, 40 s at 60°C and 35 s at 72°C. | ([Allen et al. 2001](#_ENREF_1)) | 756 bp |
| *nosZ* | *nosZ*-F  *nosZ*-1622R | CGYTGTTCMTCGACAGCCAG  CGSACCTTSTTGCCSTYGCG | 5 min at 95°C, followed by 40 cycles of 15 s at 95°C, 15 s at 60°C and 34 s at 72°C. | ([Kandeler et al. 2006](#_ENREF_3)) | 435 bp |

| **Table S2.** Concentrations of N_2_O and N_2_ produced from setups in the FeOM after 120-hour incubation.   \| Setups \| N_2_O (mmol L^-1^) \| N_2_ (mmol L^-1^) \| \| --- \| --- \| --- \| \| Control (Sterile) \| ND \| ND \| \| *B. ferrooxidans* (1×) \| 0.14 ± 0.021 \| 0.12 ± 0.052 \|   ND: not detected.  **Table S3.** Values of ^13^C/total carbon in cells of strain *B. ferroxidans* cultivated in FeOB media added with NaH^12^CO_3_ and NaH^13^CO_3_.   \| Setups \| Value \| \| --- \| --- \| \| Cells in NaH^12^CO_3_-added medium \| 0.0107 ± 0.0000100 \| \| Cells in NaH^13^CO_3_-added medium \| 0.0134 ± 0.0000770* \|   ***** indicate significant difference between the treatments at *P* < 0.05 using independent samples t-test. |
| --- | --- | --- | --- | --- | --- | --- | --- | --- | --- | --- | --- | --- | --- | --- | --- |
| **Table S4.** SOD activity of cells in two setups.   \| Setups \| SOD activity (U mg^-1^ protein) \| \| --- \| --- \| \| Cells from R2A \| 13.22 ± 1.68 \| \| Cells from ANDFO \| 38.75 ± 5.45* \| |

*indicate significant difference between the treatments at *P* < 0.05 using independent samples t-test.

1. **Supplementary References**

Allen, A. E., Booth, M. G., Frischer, M. E., Verity, P. G., Zehr, J. P., and Zani, S. (2001). Diversity and detection of nitrate assimilation genes in marine bacteria. *Appl. Environ. Microbiol.* 67, 5343-5348. doi: 10.1128/AEM.67.11.5343-5348.2001.

Conrad, R., Klose, M., and Claus, P. (2000). Phosphate inhibits acetotrophic methanogenesis on rice roots. *Appl. Environ. Microbiol.* 66, 828-831. doi: 10.1128/AEM.66.2.828-831.2000.

Kandeler, E., Deiglmayr, K., Tscherko, D., Bru, D., and Philippot, L. (2006). Abundance of *narG*, *nirS*, *nirK*, and *nosZ* genes of denitrifying bacteria during primary successions of a glacier foreland. *Appl. Environ. Microbiol.* 72, 5957-5962. doi: 10.1128/AEM.00439-06.

Miyatake, F., and Iwabuchi, K. (2005). Effect of high compost temperature on enzymatic activity and species diversity of culturable bacteria in cattle manure compost. *Bioresour. Technol.* 96, 1821-1825. doi: 10.1016/j.biortech.2005.01.005.

Molstad, L., Dorsch, P., and Bakken, L. R. (2007). Robotized incubation system for monitoring gases (O_2_, NO, N_2_O and N_2_) in denitrifying cultures. *J. Microbiol. Meth.* 71, 202-211. doi: DOI: 10.1016/j.mimet.2007.08.011.

Zhang, H., Wang, H., Yang, K., Sun, Y., Tian, J., and Lv, B. (2014). Nitrate removal by a novel autotrophic denitrifier (*Microbacterium* sp.) using Fe(II) as electron donor. *Ann. Microbiol.* 65, 1069-1078. doi: 10.1007/s13213-014-0952-6.

Zhou, G. W., Yang, X. R., Marshall, C. W., Li, H., Zheng, B. X., Yan, Y., et al. (2017). Biochar addition increases the rates of dissimilatory iron reduction and methanogenesis in ferrihydrite enrichments. *Front. Microbiol.* 8, 589. doi: 10.3389/fmicb.2017.00589.

Zhou, G. W., Yang, X. R., Sun, A. Q., Li, H., Lassen, S. B., Zheng, B. X., et al. (2019). Mobile Incubator for Iron(III) Reduction in the Gut of the Soil-Feeding Earthworm Pheretima guillelmi and Interaction with Denitrification. *Environ. Sci. Technol. Lett.* doi: 10.1021/acs.est.8b06187.
